# Further Observations on the Existence of Cellulose in Man

**Published:** 1854-06

**Authors:** R. Virchow


### *Further Observations on the Existence of Cellulose in Man.* By R. Virchow. (Virchow's Archiv., VI. p. 269.)

(Continued from May Number of the Examiner.)

Since my first communication on the presence of cellulose in the nervous centres, I have had repeated opportunities of verifying my observations. Prof. Rokitansky, too, has had the kindness to communicate to me, that he has satisfied himself of the accuracy of my statements with regard to the bodies found in the ependyma. He adds further, that he had previously met with similar bodies in an atrophied optic nerve, and in the grayish transparent fluid, which, when effused between the columns of the spinal cord, or of the medulla oblongata, causes atrophy of the medullary substance; he has also seen them repeatedly, and of large size, in rachitic bones. He was, of course, at that period not able to obtain the characteristic reaction, but he mentions, that these bodies always seemed remarkable to him on account of their different appearance from other laminated bodies, and of their dissolution when heated in the fluid containing them, whether pure or diluted with water. Their ready solubility in ether reminded him of fat, but the ease with which water dissolved them, rendered their oleaginous nature improbable. He goes on to state that the process of their dissolution differs to a certain extent from that of amylum-corpuscles. They swell up like these, but do not crack, when compressed, and when expanded to a certain size, they suddenly disappear. In all the tissues in which he found them, they were imbedded in a stroma of fibrous tissue.

I mention these interesting statements, as well as those just received from Prof. Luschka,* with great pleasure, for they may form starting points for future researches. In all probability, our knowledge of the prevalence of these bodies will be gradually increased. I am myself at present able to connect them with an affection but little understoood; a disease of the spleen, comparatively unknown, and which, though not frequent, is by no means a rare affection. It is mentioned as "lardaceous" or "waxy" spleen, (Wachsmilz) and is mainly dependant on a peculiar degeneration of the Malpighian follicles. Whilst the whole spleen increases in size and firmness, and becomes at the same time more anæmic, we find in the external portions of the contents of the follicles in question, a homogeneous, transparent, partly colored, partly grayish or yellowish zone, which increases until the whole contents of the follicle have degenerated into a slightly prominent corpuscle, of larger size than the original follicle, and exhibiting in a transverse section a viscid gelatinous appearance. Already Christensen (Copenh. Ugeskrift 1854, N. 8, or Oppenheim's Zeitschrift 1845, Sept.) compared these corpuscles to the granules of sago, seen

* Prof. Luschka discovered similar bodies in the ganglion of Gasser.—Tr.

swimming in soup, by no means a bad comparison. In the interior of these "gelatinous granules" we generally observe a white centre, the unaltered remains of the contents of the follicles.

I formerly considered these corpuscles as colloid, (see Vol. I. of this Archiv.) for I found that they were rendered pale by acetic acid, and that the addition of the ferrocyanuret of potassium produced on them a granular precipitate. Sulphuric acid, especially if hot, colored these bodies yellow, and the subsequent addition of ammonia produced the peculiar orange bluish-red color belonging to the salts of xantho-proteic acid. It seemed, therefore, reasonable to consider them either a fibrinous exudation, or an albuminous degeneration. But the exact relation of this to the other elementary degenerations was always a matter of doubt to me, (see Vol. IV. of this Archiv.) Recently I had occasion to examine a spleen thus diseased, and an accurate investigation of these little granules reminded me strongly of the corpora amylacea of the brain. They have not, it is true, their concentrically laminated appearance, but they possess the same pale, slightly opaque yet shining, and apparently soft structure. They are generally round or slightly angular, mostly homogeneous, and larger than the usual lymph corpuscles in the interior of the follicles. They lie closely together, and in laminæ, but so arranged, that the addition of sulphuric acid brings unchanged nuclei into view, evidently belonging to a fine intermediate network. On adding a watery solution of iodine, an intense yellowish red color, such as I had never before seen, was observable, and this on the addition of sulphuric acid, changed into a deep violet. The reaction was quicker than in the ependyma corpuscles; on allowing much sulphuric acid to act, a dark brownish red was produced; a little acid made the blue or violet color very perceptible. The duration of the color was in either case but temporary. The blue mass gradually became of a lighter hue, and left ultimately but a simple yellowish appearance. The great softness of these bodies seemed, therefore, to accelerate not only the appearance, but the disappearance of the color.

What I had found by a micro chemical examination, was easily verified by an analysis on a larger scale. Some of the bodies taken out of the tissue of the spleen, and treated with iodine and sulphuric acid, allowed the reaction to be well studied, even with the unassisted eye.

As this condition of the spleen is not frequently enough met with here, to make it advisable to wait for fresh specimens, I examined such as had been kept in alcohol in our pathological collection, and found that even in those that had been thus preserved for years, the reaction was readily obtained, although not with the same purity of color, observed in recent preparations. I discovered, further, another important property, which clearly demonstrates the peculiar composition of these bodies, viz., the length of time they are able to withstand decomposition. I wished to try the experiment of isolating these granules by a continued maceration, similar to the isolation of the granules in the preparation of starch, and, for this purpose, exposed the spleen to a steadily flowing current of water. This has acted on it now for three weeks, and the granules are as yet perfectly preserved. The longer they have been subjected to the current of water, the more decidedly is the reaction exhibited, so that I am now able to produce in the corpuscles a much purer and more distinct blue, than I have hitherto succeeded in obtaining. If by this process we should be able to isolate these bodies, enough of material can thus be collected for a more complete chemical investigation, and this alone would make the discovery of this method valuable. But the granules may be obtained, although not as pure, without the aid of this process, for they are readily isolated from the surrounding tissues. We will soon, therefore, I trust be able to determine whether they are devoid of nitrogen, or if they contain albuminous elements, as the formation of xantho-proteic acid seems to indicate. These particles, it is true, might be derived from the surrounding tissue or fluid; and this is not improbable, if we consider the action of acetic acid, and of ferrocyanuret of potassium on them; since in follicles thus treated, the deposit takes place, evidently, in the interstitial tissue, and not on their surface. Yet the possibility of their corpuscular origin cannot be denied, as they are evidently derived from nitrogenized substances.

To Schrant (Prijisverhandeling orer de goed-en kroaadaardige gezwellen Amsterdam, 1851, p. 291) belongs the credit of having been the first to draw attention to the origin of these bodies.

For although he wrongly denominates the disease *waxy* or *colloid* spleen, his description and drawings of the corpuscles are quite accurate. It is by a gradual transformation of the lymph corpuscles, generally contained in the follicles of the spleen, that these bodies are formed; the most external layers becoming at first homogeneous, and the whole corpuscle solid. Thus though in this disease we have not, as supposed, a colloid cell-metamorphosis, we are justified in speaking of it as a *cellulose metamorphosis of cells.*

How prevalent this metamorphosis, it is at present difficult to determine. Wedl (Grundzüge der Pathologischen Histologie) figures similar laminated colloid corpuscles, taken from the intermuscular tissue of an hypertrophied heart. Schrant (Tijdschrift. der Nederl. Maatschapheiy, 1852, July, p. 260, fig. iv.) has drawn concentrically laminated colloid corpuscles from the optic nerve of an amaurotic patient. Fresh investigations are required to prove the real nature of these bodies. I have examined recently the so-called colloid of the thyroid gland, of the ovaries, and kidney, also the elements of intervertebral cartilage, and several lardaceous livers, but in vain; the corpuscles they contained were not cellulose-corpuscles. The term colloid must, however, be used with greater caution. After having separated from it the mucous tissue, (Schlmeingewebe) the cellulose, and certain albuminous masses, such as seen in the prostate gland, we will perhaps be able to find further distinctions.

As cellulose has thus been found in an important pathological process, it might not be inadvisable to say a few words of the peculiar disease of the spleen in which it occurs. Christensen mentions "waxy spleen" in connection with albuminuria, complicated with hydropic effusion and Bright's disease. This is indeed very frequent. This condition of the spleen is generally attended with a cachectic state of the system of long duration, and with disease of the liver and kidney. I have most frequently seen it accompanying chronic ulcerative processes, as in caries, tuberculosis, and dysenteric affections of the intestines, but also in the cachectic condition of the system which occurs with nephritis after scarlet fever. It appears as if this were always followed by a permanent alteration in the structure of the spleen, but I must confess that this is to me a very obscure point. DAC.

